# Remaining Life Prediction of Shielding Sleeves Based on Data Augmentation and Hybrid Models

**DOI:** 10.3390/s26113367

**Published:** 2026-05-26

**Authors:** Xin Zhang, Xuewei Xiang, Hui Li, Nengqing Liu, Zhi Chen

**Affiliations:** 1 State Key Laboratory of Power Transmission Equipment Technology, School of Electrical Engineering, Chongqing University, Chongqing 400044, China; xxxz0516@163.com (X.Z.); xueweixiang@cqu.edu.cn (X.X.); lnq_cqu@163.com (N.L.); 2 National Key Laboratory of Nuclear Reactor Technology, Nuclear Power Institute of China, Chengdu 610213, China; chenzhinpic@126.com

**Keywords:** main pump motor, shielding sleeve, data-driven, hybrid model, Monte Carlo simulation

## Abstract

Remaining useful life (RUL) prediction is a critical procedure to avoid catastrophic failure of shielding sleeves and prevent nuclear safety risks. Affected by the structural characteristics and service conditions of shielding sleeves, it is difficult to obtain sufficient full-life-cycle actual degradation data, which greatly restricts the training and application of data-driven prediction models. This paper proposes a remaining useful life prediction method for shielding sleeves based on data augmentation and a hybrid model. Firstly, starting from the physical failure mechanisms of two typical failure modes of shielding sleeves, namely bulging and wear. Secondly, based on the analytical models of typical failures of shielding sleeves, a degradation data augmentation method using Monte Carlo simulation is proposed to address the problem of missing full-life-cycle degradation data. Finally, a hybrid RUL prediction model for shielding sleeves based on Stacking ensemble learning is presented, which integrates the advantages of physical degradation models and deep learning methods. Experimental verification is carried out through multiple sets of degradation datasets with different failure modes. The root-mean-square error (RMSE) of the proposed prediction method can reach a minimum of 0.0058, and the mean absolute error (MAE) can reach a minimum of 0.0044. The prediction accuracy is superior to that of single models, which verifies the prediction performance and engineering applicability of the proposed method.

## 1. Introduction

The nuclear reactor coolant pump is one of the core pieces of equipment in nuclear power plants. As the only key device operating continuously for a long term in nuclear reactors, it undertakes the vital task of driving coolant circulation [1,2]. Among them, the shielding sleeve serves as one of the critical components of the main pump, and its integrity is directly related to the normal operation of the primary circuit of the entire nuclear power plant. During operation, the shielding sleeve is subjected to the combined effects of high-temperature and high-pressure coolant, electromagnetic force and operating condition variations, making it prone to failure modes such as bulging deformation, wear and rupture. Ensuring online degradation assessment and prediction of the shielding sleeve is essential to guarantee the long-term stable operation of the system [3,4]. Nevertheless, the highly compact internal structure of the shielding sleeve makes it extremely difficult to install additional sensors for direct acquisition of characteristic data, and sufficient full-life-cycle degradation data of the shielding sleeve are hard to obtain, which greatly restricts the training and application of data-driven prediction models. In addition, the complexity of the service conditions of the shielding sleeve also poses a significant challenge to traditional life prediction methods.

At present, studies on the degradation of shielding sleeves are mainly conducted from the perspectives of steady-state performance and structural stability. In [5], the forced deformation response of shielding sleeves made of different materials was analyzed by calculating the linear density of electromagnetic force inside the shielding sleeve. Deokjoo Kim [6] adopted the finite element method to investigate the structural stability of shielding sleeves and introduced local geometric imperfections to study the influence of initial imperfections on structural stability. Lo Frano [7] explored the effect of imperfections on the buckling of externally pressurized thin-walled shells, established corresponding models for simulation and calculation, and investigated the influences of material properties and external pressure conditions on the structural stability of shells, finally verifying the accuracy of structural stability results obtained via elasto-plastic finite element simulation analysis. However, none of the existing studies has considered the time-dependent evolution law of shielding sleeve failure, and it remains difficult to obtain sufficient full-life-cycle degradation data of shielding sleeves, which greatly restricts the training and application of data-driven prediction models.

In terms of life prediction modeling, physics-based life prediction can provide prediction results with minimum uncertainty when the physical mechanism is complete. Forman [8] proposed a modified Paris-Erdogan model to make it more suitable for conditions with high stress intensity factors. Oppenheimer [9] utilized the Forman formula to predict the service life under rotor crack propagation. In mechanical systems, cumulative damage models are employed to describe the damage accumulation process of materials under long-term loading and environmental effects, and are widely used in fatigue crack propagation [10]. The nonlinear degradation process can be further considered by adding a nonlinear cumulative index to the cumulative damage term [11]. However, it is quite challenging to establish an accurate physical model for the degradation process of complex systems, and physical models are highly dependent on the specific characteristics of the research object, making it difficult to achieve generalization.

With the rapid development of computer and intelligent technologies, deep learning models have exhibited broad application potential in the field of equipment life prediction. Roodschild [12] constructed an improved feedforward neural network by adding a constant to the original sigmoid activation function to solve the vanishing gradient problem. Ma [13] adopted the particle swarm optimization algorithm to optimize the initial weights and thresholds of the FNN, improving the stability and learning efficiency of the network. Hochreiter [14] proposed the long short-term memory network, which selectively memorizes information through forget and input gates and effectively solves the long-term prediction problem. Cho [15] put forward the gated recurrent unit by integrating the forget and input gates. Graves [16] proposed combining forward LSTM and backward LSTM, enabling the model to utilize both past and future information and improving the prediction capability. In addition, a variety of techniques have been developed in the field of deep learning, such as generative adversarial networks [17], auto encoders [18], and attention mechanism-based Transformers [19], which have shown unique advantages in sequence modeling, data augmentation, feature extraction and other fields. However, in the specific application domain of nuclear coolant pump shielding sleeves, these pure data-driven frontiers face critical applicability challenges. Firstly, Transformers rely heavily on massive datasets to optimize complex attention matrices, making them highly susceptible to overfitting when actual full-life-cycle data is extremely scarce. Secondly, while GANs and diffusion models excel at generating realistic distributions, they cannot guarantee that the synthetic degradation trajectories strictly obey the physical bounds of material creep and wear mechanisms, which is an unacceptable risk for nuclear safety. Therefore, instead of these purely data-driven generators, this paper deliberately adopts a Monte Carlo simulation driven by explicit physical constitutive equations to ensure the rigorous physical interpretability of the augmented data.

Based on the above analysis, this paper proposes a remaining useful life prediction method for shielding sleeves based on data augmentation and a hybrid model. A creep constitutive model for bulging and an analytical model for contact wear of shielding sleeves are established to clarify the mapping relationship between operating condition loads and degradation evolution laws. On this basis, a degradation data augmentation method based on Monte Carlo simulation is proposed to solve the problem of missing full-life-cycle degradation data. We propose a hybrid RUL prediction model based on Stacking ensemble learning. The novelty lies in utilizing a Particle Filter (PF) to make the physical degradation model dynamically adaptive online, serving as a base learner to capture explicit macro-trends, while deploying an LSTM-CNN to extract unmodeled micro-nonlinearities. This specific architecture achieves complementary advantages between dynamic physical transparency and deep learning adaptability under severe data scarcity.

## 2. Data Augmentation Method Based on Monte Carlo Simulation

### 2.1. Creep Constitutive Model for Bulging of Shielding Sleeves

During the operation of the canned motor reactor coolant pump, the coolant flows at a high speed inside the shielding sleeve to provide radial support and thermal load. Based on the collection and analysis of historical service data of the main pump, within one overhaul cycle, the shielding sleeve has to withstand long-term full-power operation with a temperature of 273 °C and a coolant loop system pressure of 15.0 MPa, as well as multiple transient operating conditions including power ramp-up and ramp-down, switching between high and low speeds of the main pump, and load rejection. Under extreme transient conditions, its peak pressure can reach 17.06 MPa and the peak temperature exceeds 282 °C. Under the long-term combined action of the aforementioned high temperature, high pressure and alternating loads, the shielding sleeve is highly prone to material creep accumulation, which eventually leads to bulging deformation.

**Figure 1:**
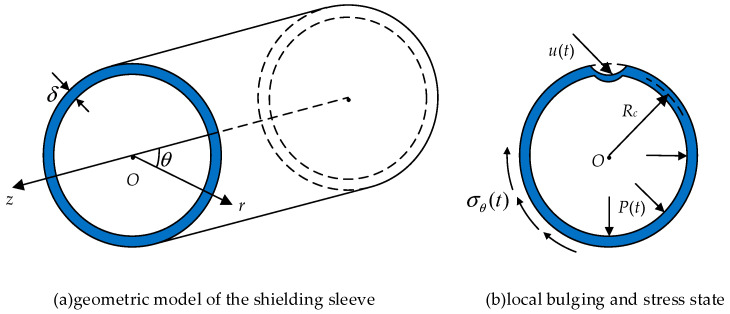
Schematic Diagram of the Bulging Mechanism of the Shielding Sleeve.

To accurately characterize this degradation process, the shielding sleeves are simplified as a thin-walled cylindrical structure subjected to internal pressure, as shown in Figure 1.

Let the mean radius of the shielding sleeves be *R_c_* and the wall thickness be *δ*. Under the action of the internal coolant pressure *P*(*t*), the main principal stress generated in the wall of the shielding sleeves is mainly the hoop stress; according to the thin-walled pressure vessel theory, its hoop stress $\sigma_{\theta }(t)$ can be expressed as:

(1) $$\sigma_{\theta }(t)=\frac{P(t)\cdot R_{c}}{\delta }$$

In high-temperature service environments, shielding sleeve materials such as Hastelloy exhibit significant creep characteristics. In this paper, the classical Norton-Bailey steady-state creep model is adopted to describe the strain rate evolution of shielding sleeves under high temperature and high pressure:

(2) $${\dot{\varepsilon }}_{cr}(t)=A\cdot \sigma_{\theta }(t{)}^{n}\cdot \exp\left(-\frac{Q}{R_{g}\cdot T(t)}\right)$$

where ${\dot{\varepsilon }}_{cr}(t)$ is the creep strain rate at time *t*; *A* and *n* are the creep constants associated with material properties; *Q* is the creep activation energy of the material; *R_g_* is the universal gas constant; and T(t) is the absolute temperature of the shielding sleeves at time *t*.

The evolution law of the bulging failure of the shielding sleeves is characterized by the variation law of the radial deformation with time, and the geometric relationship between the strain and the radial deformation u(t) can be expressed as:

(3) $$\varepsilon_{cr}(t)=\frac{u(t)}{R_{c}}$$

Substituting the formula for hoop stress into the creep constitutive equation and integrating with respect to time yields the analytical equation describing the time-dependent evolution of the radial deformation caused by the bulging of the shielding sleeves:

(4) $$u(t)=R_{c}\int_{0}^{t}A{\left[\frac{P(\tau )\cdot R_{c}}{\delta }\right]}^{n}\exp\left(-\frac{Q}{R_{g}\cdot T(\tau )}\right)d\tau$$

Since the shielding sleeve is installed in the motor air gap, the radial deformation displacement u of the bulge must not exceed the air gap length. Therefore, the failure threshold of the bulge is defined as 90% of the air gap length, meaning that when the radial deformation displacement reaches the threshold, the shielding sleeve is regarded as completely failed and the main pump motor cannot continue to operate.

This analytical model establishes a mathematical mapping from microscopic material creep to macroscopic component bulging deformation, and can directly take time-series inputs of time-fluctuating temperature and pressure, thus performing data augmentation for the subsequent bulging dataset of the shielding sleeves.

### 2.2. Analysis Model for Contact Wear of Shielding Sleeves

Under ideal hydrodynamic lubrication conditions, the main pump rotor is supported by water-lubricated bearings, and a stable coolant liquid film exists between the rotor shielding sleeve and the stator shielding sleeve without any mechanical contact between them. However, according to the actual operating conditions of the main pump during the overhaul cycle, the motor has to undergo multiple transient processes such as reactor startup, reactor shutdown, and switching between high and low speeds of the main pump during service. During low-speed operation or transient speed change stages, it is difficult for water-lubricated bearings to form a complete hydrodynamic lubricating film, and the bearings operate under boundary lubrication or mixed lubrication conditions, which inevitably leads to continuous wear of the radial sliding bearings. With the gradual accumulation of bearing wear, the radial support clearance of the rotor system increases, and the rotor experiences radial eccentricity under the action of dead weight, fluid excitation force or unbalanced magnetic tension. When the radial eccentric displacement of the rotor exceeds the initial design clearance between the stator and rotor shielding sleeves, the high-speed rotating rotor shielding sleeve will directly undergo dynamic-static rub-impact with the inner wall of the stator shielding sleeve.

**Figure 2:**
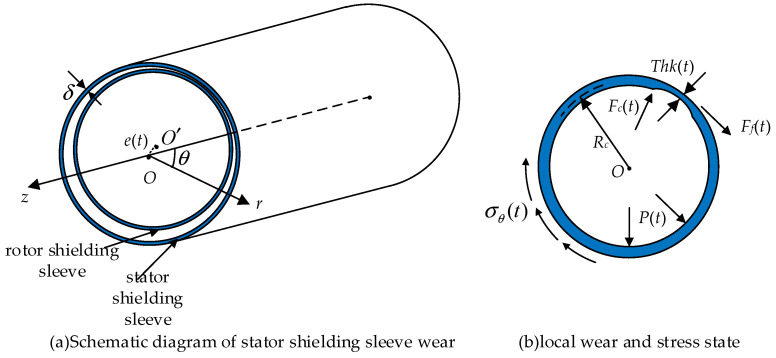
Schematic diagram of the wear mechanism of the shielding sleeve.

Under such severe friction conditions, owing to the extremely small wall thickness of the stator shielding sleeve, the surface asperities of the material undergo plastic yielding and fracture under shear stress and spall to form abrasive particles, representing typical adhesive wear and abrasive wear. The continuous increase in wear depth will eventually lead to the thinning of the shielding sleeve wall thickness until perforation failure occurs. The schematic diagram of the wear failure mechanism of the shielding sleeve is shown in Figure 2.

To establish a computable analytical model, this paper sets the following wear degradation scenarios based on the cascading failure logic between components: Firstly, during the bearing degradation stage, the shielding sleeves remain in a healthy condition, but the radial water-lubricated guide bearings suffer from a time-increasing wear volume $\omega_{b}$(*t*) due to reactor startup and shutdown as well as high-low speed switching, resulting in synchronous eccentric displacement of the rotor, where $e_{0}$ denotes the initial installation eccentricity; next is the mild wear stage, and rub-impact is initiated when the eccentric displacement e(t) reaches the initial radial clearance $c_{0}$ between the rotor and stator shielding sleeves, at which point the contact stress is low and the wear proceeds slowly; finally, the severe wear stage is entered: with further wear of the bearings, the extrusion of the rotor against the stator shielding sleeves is intensified, the normal contact force increases sharply, local and continuous wear bands form on the inner wall of the stator shielding sleeves, and the wall thickness decreases rapidly until the residual thickness falls below the safety threshold for pressure bearing.

Based on the above scenario, this paper introduces the Archard theory to establish an analytical model for the wear degradation of the stator shielding sleeve. The Archard equation describes the linear relationship between the sliding contact volume wear, the contact load and the sliding distance at the macroscopic scale. Defining the local wear depth of the stator shielding sleeve as h(t), its evolution rate with time can be expressed according to the Archard wear rate equation as:

(5) $$\frac{dh(t)}{dt}=k_{w}\cdot p_{c}(t)\cdot v_{s}(t)$$

where $k_{w}$ is the specific wear rate of the material, which is generally equal to the ratio of the wear coefficient *K* to the material hardness *H*; $p_{c}(t)$ is the transient contact pressure between the rotor and stator shielding sleeves at time *t*; and $v_{s}(t)$ is the relative sliding linear velocity.

The relative sliding speed $v_{s}(t)$ is determined by the outer diameter *R*_r_ of the rotor and can be expressed as:

(6) $$v_{s}(t)=\omega (t)\cdot R_{r}$$

where ω(t) is the rotor angular velocity, which needs to be assigned in segments according to the scheduling timetables under different working conditions in the historical service data.

The contact pressure $p_{c}(t)$ depends on the extrusion force $F_{N}(t)$ of the rotor on the stator and the actual contact area $A_{c}$. Assuming that the rotor system has an equivalent radial bending stiffness $K_{r}$, the normal contact force can be simplified as a linear spring model when the rotor eccentricity *e*(*t*) exceeds the initial radial clearance $c_{0}$ between the rotor and stator shielding sleeves:

(7) $$F_{N}(t)=\left\{\begin{array}{c} 0,e(t)\leq c_{0}\\ K_{r}\cdot (e(t)-c_{0}),e(t)>c_{0} \end{array}\right.$$

The contact pressure $p_{c}(t)$ can be expressed as:

(8) $$p_{c}(t)=\frac{F_{N}(t)}{A_{c}}$$

Substituting Equations (6)–(8) into Equation (5) and integrating with respect to time yields the cumulative wear depth h(t) of the inner wall of the stator shielding sleeve at time *t*:

(9) $$h(t)=\int_{t_{c}}^{t}k_{w}\cdot \left[\frac{K_{r}\cdot (e(\tau )-c_{0})}{A_{c}}\right]\cdot \omega (\tau )R_{r}d\tau$$

where $t_{c}$ is the rub-impact trigger time, that is, the critical moment when e(t) = $c_{0}$.

Define the remaining thickness Thk(t) of the shielding sleeve evolving over time as:

(10) Thk(t)=δ−h(t)

When the residual thickness Thk(t) is less than the critical thickness *δ*_min_ required to satisfy the pressure-bearing requirement, wear failure is determined. This analytical model can take the speed step of the main pump as an operating condition factor and clearly characterize the nonlinear degradation trajectory of the shielding sleeve from a healthy state to wear occurrence and then to aggravated wear, laying a foundation for data augmentation of the subsequent shielding sleeve wear dataset.

### 2.3. Data Augmentation and Enhancement Under Variable Operating Conditions

Although the analytical model reveals the degradation law of the shielding sleeves, using only a single deterministic operating condition input yields only one definite degradation curve, which cannot meet the training requirements of deep learning models for large-scale and diverse samples. In actual operation, affected by power grid load fluctuations, sensor measurement errors and material processing variability, the operating parameters and material properties of the main pump show a certain degree of randomness.

**Figure 3:**
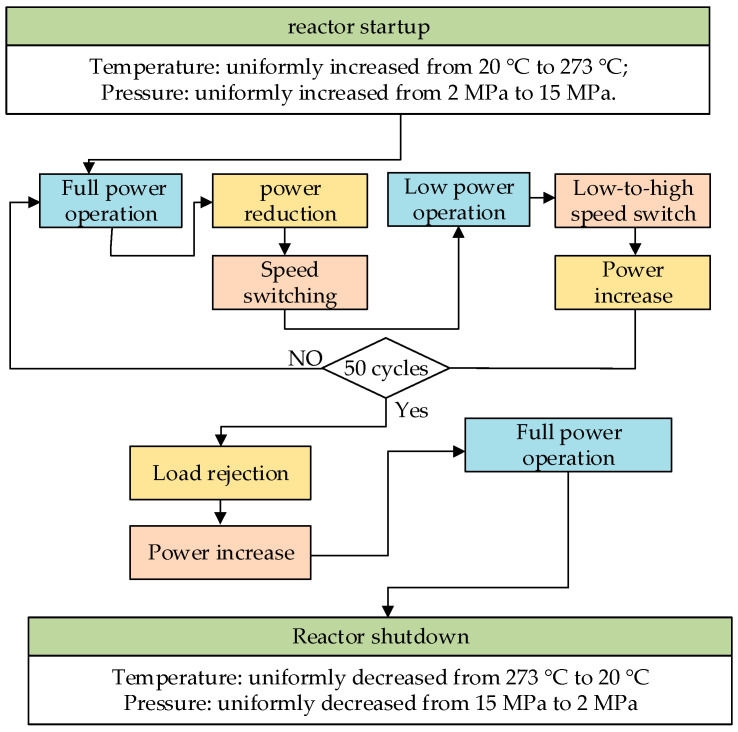
Schematic diagram of variable operating conditions.

Therefore, this paper introduces the Monte Carlo simulation method to set the probability distribution of input operating conditions and material parameters, and simulates the uncertainty in the actual service environment through a large number of random samplings. According to the historical service conditions of the main pump motor, one overhaul cycle is set as 30 major operating condition cycles, and the variable operating conditions within one major cycle are shown in Figure 3.

**Figure 4:**
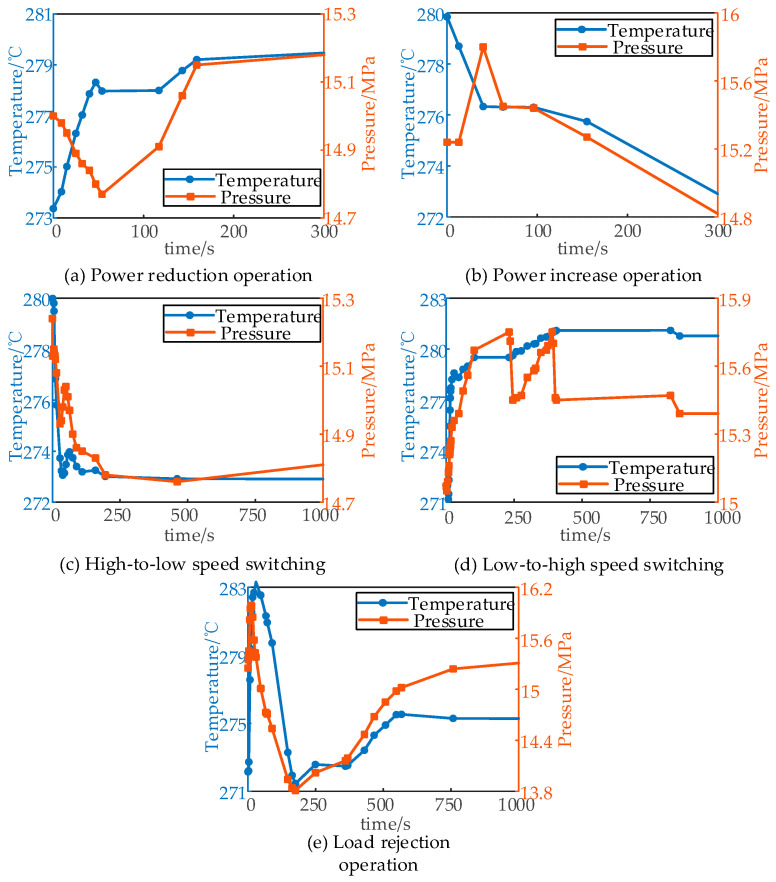
Temperature and pressure curves of the shielding sleeve.

The temperature of the shielding sleeve and the pressure of the coolant circuit system under different working conditions are plotted in Figure 4.

Based on the operating data within the maintenance cycle, the distribution of the model’s random variables is defined. Considering the fluctuations in actual working conditions, it is set that the working condition parameters follow a normal distribution. Considering the material differences of shielding sleeves from different batches, the creep constant *A* of Hastelloy is set to follow a uniform distribution, and the initial installation clearance *c*_0_ between the rotor and stator shielding sleeves is set to follow a normal distribution.

**Figure 5:**
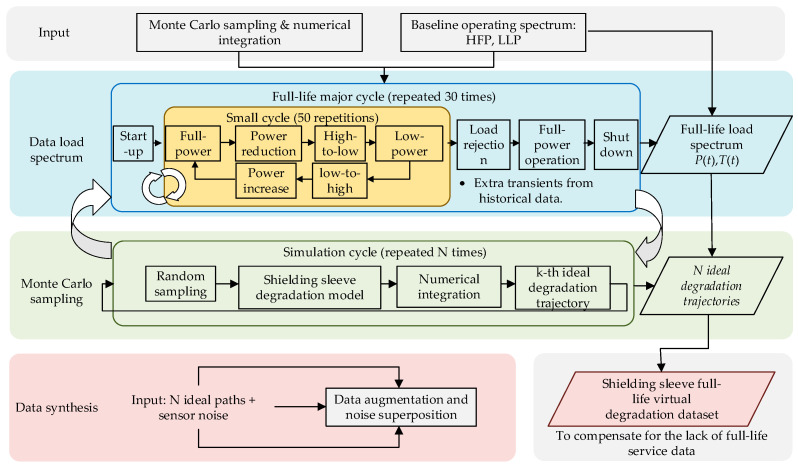
Flowchart of Data Augmentation Method Based on Monte Carlo.

Based on the above model parameter distribution settings, the Monte Carlo data augmentation process is shown in Figure 5, which mainly includes load spectrum splicing, random sampling and calculation, and monitoring data synthesis.

**Figure 6:**
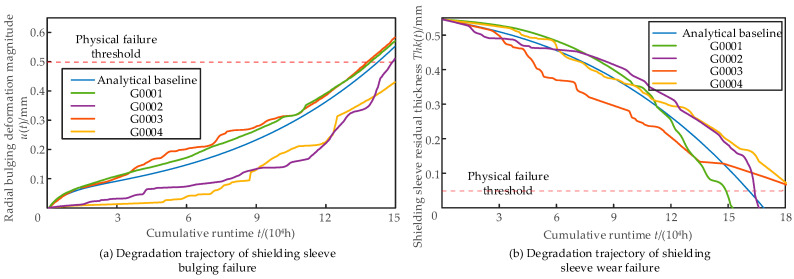
Degradation trajectories of shielding sleeves under different failure modes.

Data augmentation for the bulging and wear degradation datasets of shielding sleeves is performed based on the Monte Carlo method. To verify that the subsequent prediction method has strong generalization ability for the life prediction of shielding sleeves under different operating conditions, four bulging degradation trajectories and four wear degradation trajectories of shielding sleeves are randomly selected as data samples for subsequent life prediction. The bulging dataset of shielding sleeves is numbered G001–G004, and the wear dataset is numbered M001–M004. The maximum time length of the datasets is 1.5 × 10^5^ h with a time step of 1 h. The degradation trajectories of shielding sleeves are shown in Figure 6.

## 3. Hybrid Model for Predicting the Service Life of Shield Sleeves Based on Ensemble Learning

### 3.1. Degradation Model with Parameter Adaptive Update Based on Particle Filter

During long-term actual operation, the main pump undergoes reactor startup and shutdown, power raising and lowering, as well as various transient disturbances, resulting in dynamic alternating operating temperature and pressure. In addition, the microscopic aging law of the material drifts with the increase in service time. Therefore, the fixed-parameter model fitted offline based on historical data can hardly accurately characterize the actual real-time degradation state of the shielding sleeves. The introduction of a filtering algorithm to optimize the model parameters can be considered to further improve the applicability and robustness of the model.

In terms of filtering algorithm selection, traditional Bayesian filtering obtains the posterior distribution using prior knowledge, and its computational complexity increases sharply with the growth of state dimensions, which may lead to difficulties in solving problems in complex data samples. As a form of Bayesian filtering, Kalman filtering can effectively solve linear Gaussian problems. However, in practice, systems or devices often face more complex scenarios and are not based on linear assumptions. To eliminate prediction errors caused by individual differences and time-varying operating conditions, the Particle Filter (PF) algorithm is introduced in this section. Compared with the Extended Kalman Filter (EKF), the PF algorithm is based on the Monte Carlo principle and Bayesian estimation theory, free from the limitations of linearization errors and Gaussian distribution assumptions, and thus has significant advantages in handling the highly nonlinear degradation model of shielding sleeves. By continuously incorporating the latest online monitoring or maintenance data, the PF algorithm can realize adaptive dynamic update of the parameters of the degradation model.

The state-space model of PF consists of the state transition equation and the observation equation. Considering the slowly time-varying characteristic of parameter degradation during actual equipment operation, the state transition equation of the PF algorithm can be expressed as:

(11) $$\theta_{k}=\theta_{k-1}+\omega_{k-1}$$

where $\theta_{k}$ and $\theta_{k-1}$ are the model parameter vectors at time $t_{k}$ and $t_{k-1}$; $\omega_{k-1}$ is the process noise vector, which is generally assumed to follow a normal distribution with zero mean ω∼N(0,Q), where ***Q*** is the process noise covariance matrix that characterizes the uncertainty of parameter drift.

The observation equation describes the mapping relationship between the hidden state parameters and the degradation index of the shielding sleeve:

(12) $$z_{k}=H(t_{k},\theta_{k})+\nu_{k}=a_{k}\exp(b_{k}t_{k})+c_{k}\exp(d_{k}t_{k})+\nu_{k}$$

where $z_{k}$ is the actual observation of the degradation state of the shielding sleeve at time $t_{k}$; *H*($t_{k}$, $\theta_{k}$) is the nonlinear observation function; *v_k_* is the measurement noise, which is assumed to be an independently and identically distributed Gaussian random process $\nu_{k}∼N(0,R)$, and *R* is the observation noise variance. Crucially, the observation function *H*($t_{k}$, $\theta_{k}$) is not an arbitrary mathematical construct but strictly corresponds to the analytical degradation models derived in Section 2. Specifically, for the bulging failure mode, *H*($t_{k}$, $\theta_{k}$) utilizes the analytical solution of radial deformation *u*(*t*) presented in Equation (4); for the wear failure mode, it corresponds to the residual thickness Thk(t) in Equation (10). Its physical interpretation is the deterministic mapping from the hidden micro-degradation parameters $\theta_{k}$ to the macroscopically measurable degradation indicators.

To ensure reproducibility and tracking stability, the number of particles is set to *N* = 1000, which provides an optimal balance between estimation accuracy and computational efficiency for the low-dimensional parameter space in this study. A common issue in PF is particle degeneracy, where most particles gain negligible weights over time. To address this, the Effective Sample Size *N*_eff_ is monitored at each time step. When *N*_eff_ falls below the threshold of *N*/2, a Systematic Resampling strategy is triggered. Systematic resampling is employed because it minimizes the variance of the particles and operates efficiently with a time complexity of *O*(*N*). Regarding computational complexity, since the PF only executes sequential state prediction and weight updating for *N* particles per time step (with an *O*(*N*) complexity), its computational footprint is extremely light. Compared to the deep learning module (LSTM-CNN) which dominates the training time due to backpropagation over long sequences, the integration of the PF component does not introduce any computational bottleneck to the overall hybrid framework.

After constructing the state-space model, the Particle Filter (PF) is used to adaptively update the parameters of the shielding sleeve degradation model. The core idea of the algorithm is to approximate the posterior probability density function *p*($\theta_{k}$|$z_{1:k}$) of the system state with a set of random samples with weights. State estimation and output are performed through particle initialization, state prediction and weight update, thereby adaptively updating the model parameters.

### 3.2. Deep Learning Network Based on LSTM-CNN

**Figure 7:**
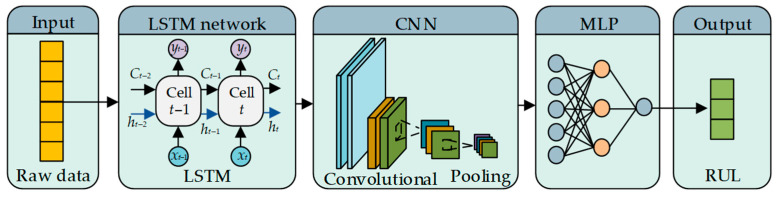
Flowchart of Deep Learning Network Based on LSTM-CNN.

The shielding sleeve degradation model based on PF parameter adaptive updating has obvious limitations in mining the implicit complex relationships in the data of complex equipment systems, while machine learning methods show significant advantages in processing high-dimensional, nonlinear sequences with long-term dependence. Long Short-Term Memory (LSTM) networks can effectively capture long-term dependencies in time-series data, and Convolutional Neural Networks (CNNs) can efficiently extract important local features from time-series data. By constructing an LSTM-CNN network, the characteristics of the two networks can be combined to improve the representation ability and prediction accuracy of the model. The constructed LSTM-CNN network framework is shown in Figure 7.

**Figure 8:**
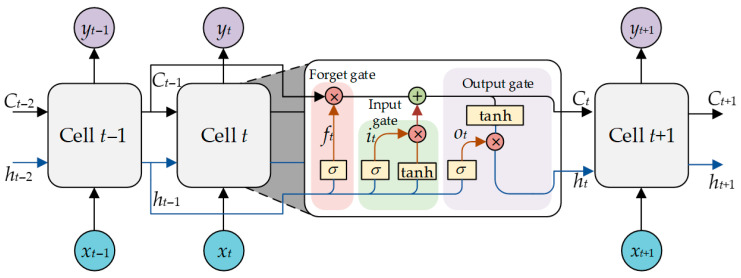
LSTM network structure.

As a special type of Recurrent Neural Network (RNN), LSTM effectively overcomes the vanishing or exploding gradient problem that conventional RNNs easily suffer from when processing long sequences by introducing a gating mechanism. It is suitable for dealing with degradation sequences as long as tens of thousands of hours, such as the degradation process of shielding sleeves. The basic structure of the LSTM network is shown in Figure 8.

The LSTM unit contains three core gating structures internally: the forget gate, the input gate, and the output gate. Let the input feature vector at time *t* be $x_{t}$, the hidden state at the previous moment be $h_{t-1}$, and the memory cell state be $C_{t-1}$. The forget gate decides which information to retain and which useless information to discard from the cell state $C_{t-1}$. at the previous moment:

(13) $$f_{t}=\sigma (W_{f}\cdot [h_{t-1},x_{t}]+b_{f})$$

The input gate and candidate memory cells determine which new information needs to be stored in the current cell state:

(14) $$\left\{\begin{array}{c} i_{t}=\sigma (W_{i}\cdot [h_{t-1},x_{t}]+b_{i})\\ {\tilde{C}}_{t}=\tan h(W_{C}\cdot [h_{t-1},x_{t}]+b_{C}) \end{array}\right.$$

Combine the results of the forget gate and the input gate to update the current memory cell state $C_{t}$:

(15) $$C_{t}=f_{t}⊙C_{t-1}+i_{t}⊙{\tilde{C}}_{t}$$

The output gate and the hidden state determine which information in the current cell state will be transmitted as the output $h_{t}$ at this moment to the next layer:

(16) $$\left\{\begin{array}{c} o_{t}=\sigma (W_{o}\cdot [h_{t-1},x_{t}]+b_{o})\\ h_{t}=o_{t}⊙\tan h(C_{t}) \end{array}\right.$$

where *σ*(·) and tanh(·) are activation functions; *W* and b are the weight matrix and bias vector of the corresponding gating structure, respectively; ⊙ represents the Hadamard product of matrix elements.

Although LSTM can effectively establish the global temporal dependence of shielding sleeve degradation, it is not sensitive enough to local feature changes caused by sudden changes in operating conditions. Therefore, a one-dimensional convolutional neural network (1D-CNN) is cascaded after the LSTM layer to enhance local features.

Let the sequence matrix of hidden states output by the LSTM layer be ***H*** = [*h*_1_, *h*_2_, …, *h*_T_], where *T* denotes the length of the time window. The 1D-CNN performs sliding operations on this sequence using one-dimensional convolutional kernels of size *K*. The convolutional output of the *j* convolutional kernel at the *i* step can be expressed as:

(17) $$c_{i}^{j}=f\left(\sum_{k=1}^{K}w_{k}^{j}\cdot h_{i+k-1}+b^{j}\right)$$

where $w_{k}^{j}$ is the weight of the *j* convolution kernel, $b^{j}$ is the bias term. After the convolution operation, to reduce the feature dimension and retain the most significant local mutation features, a max-pooling layer is connected to extract the maximum value within a given pooling window:

(18) $$p_{m}^{j}=\underset{i\in W_{m}}{max}(c_{i}^{j})$$

Through multi-scale feature extraction and dimensionality reduction by the CNN layer, the model effectively filters out redundant background noise in sensor monitoring data and strengthens the local key features that characterize abrupt changes in degradation rate. The fused feature map extracted by the LSTM-CNN module is flattened into a one-dimensional vector, which is then input into the fully connected layer composed of a Multi-Layer Perceptron (MLP) for mapping to obtain the output.

### 3.3. Hybrid Model for Life Prediction Based on Stacking Ensemble Learning

This paper proposes a hybrid model for shielding sleeve life prediction based on the Stacking strategy. By constructing a multi-level learning structure, it combines the macro trend prediction capability of physical degradation models with the micro feature capture capability of deep learning models. The meta-learner adaptively assigns prediction weights, thereby significantly improving the accuracy, robustness and generalization ability of shielding sleeve life prediction.

The degradation model and the LSTM-CNN network are taken as the two base models in the Stacking strategy, and Output 1 and Output 2 are obtained by the two base models respectively. When a time series is non-stationary, transforming it using stationarity processing methods such as differencing generally helps improve the prediction accuracy of RUL. The formula for the *D*-th order difference is as follows:

(19) $$\Delta^{D}x_{t}={\left(1-L\right)}^{D}x_{t}$$

In the formula, *x_t_* represents the sample data at time *t*, and *L* is the lag operator, defined as:

(20) $$L^{D}x_{t}=x_{t-D}$$

**Figure 9:**
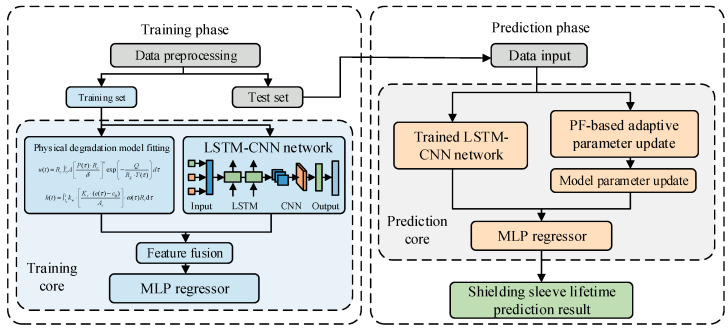
Flowchart of Lifetime Prediction Based on Stacking Ensemble Learning.

MLP is used as the meta-model to fuse the outputs of the two base models, the raw data and the difference data, so as to achieve the RUL prediction. The framework of the hybrid model proposed in this paper is shown in Figure 9.

The training adopts the standard two-stage Stacking strategy. First, the physical model and the LSTM-CNN network are independently trained and fitted. After the base models converge, their parameters are completely frozen. Subsequently, we use the outputs of the base models on the validation set to construct the feature space for the meta-learner, and independently train the MLP meta-learner. The reason for not adopting joint optimization is to prevent the deep learning model with a large number of parameters from dominating the gradients during backpropagation, thereby undermining the macro-degradation physical laws with clear interpretability captured by the physical model.

## 4. Experimental Results and Analysis

To verify the effectiveness of the proposed method, the bulging datasets G001–G004 and wear datasets M001–M004 are used for validation. The ratio of the training set to the test set for each dataset is set to 2:1 (i.e., the prediction start time is 10 × 10^4^ h). In the hybrid model, the convolutional kernel size is 5, the number of channels is 4, and the number of LSTM hidden layer units is 64. The model reads 2000-h data segments as input sequences each time and performs overlapping sliding sampling with a step size of 300 h. This approach ensures extremely high fitting accuracy while significantly reducing the number of slices, thereby shortening the computation time. Piecewise constant decay is adopted, with an initial learning rate set to 0.005 to enable the network to quickly escape local optima in the early stage of training. The learning rate is automatically halved every 15 Epochs, which ensures smooth convergence of the network during the weight fine-tuning phase in the later training stage and avoids severe fluctuations in the prediction curve. The geometric dimensions and material parameters of the shielding sleeve are shown in Table 1.

### 4.1. Experimental Results of the Parameter Adaptive Update Model

Tests were carried out using the data-augmented shielding sleeve degradation dataset. Firstly, a polynomial model for shielding sleeve degradation is established through offline fitting based on historical data, and the initial parameters of the adaptive update algorithm are determined. By continuously integrating degradation data, the PF algorithm is employed to achieve adaptive dynamic updating of the parameters of the degradation model. The initial parameters of the adaptive update algorithm are presented in Table 2.

**Figure 10:**
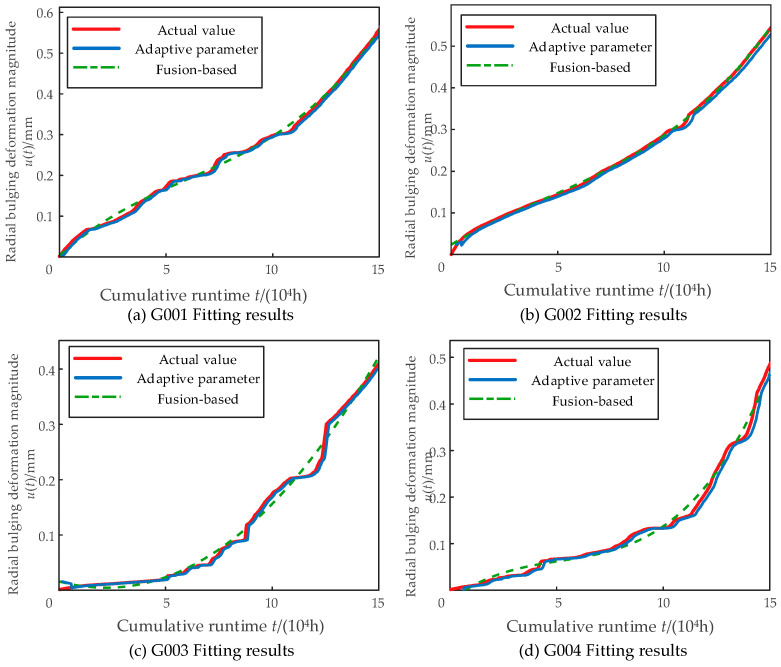
Fitting of the adaptive model for bulging degradation of shielding sleeves.

**Figure 11:**
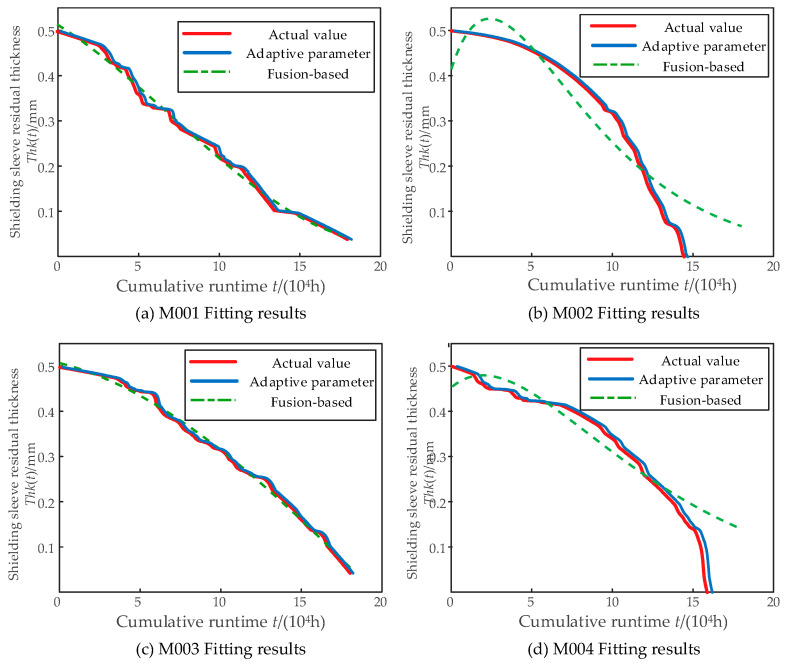
Fitting of adaptive model for wear and degradation of shielding sleeve.

The experimental effect of the bulging degradation adaptive model is shown in Figure 10, and that of the wear degradation adaptive model is shown in Figure 11.

By analyzing the fitting curve, compared with the polynomial fitting model, the degradation model based on PF parameter adaptive updating can effectively characterize the changes in the model state parameters during the shielding sleeve degradation process, and can accurately capture the random fluctuations caused by operating condition changes or measurement noise.

However, since the parameter adaptive model dynamically updates the unknown parameters of the degradation model using the latest continuously acquired data, when the actual degradation trajectory of the shielding sleeve undergoes a local abrupt change or shows an accelerating trend with increasing curvature, the algorithm has to go through the complete process of inputting new observation data and calculating the posterior state to update the driving parameters. Such a mechanism of recursively revising the prior distribution based on time series determines that the model’s response to sudden nonlinear changes inevitably has an inherent dynamic delay on the time axis.

In addition, considering that measurement data under actual working conditions are inevitably disturbed by environmental noise and sensor errors, when a real local degradation jump occurs in the system, the PF algorithm tends to conservatively attribute part of the increment to measurement noise in the first few sampling periods. Only after accumulating sufficient subsequent data points to confirm the authenticity of the accelerating trend will the model parameters shift significantly. This noise suppression mechanism greatly improves the global robustness of the model, but at the same time manifests itself macroscopically as a lag in tracking the real abrupt change. However, it also exposes a specific boundary case where the PF model momentarily deviates or fails to track perfectly: when a severe and genuine non-linear degradation jump occurs, the PF algorithm conservatively hesitates, exhibiting a quantitative dynamic lag of approximately 500 to 1500 h before its parameters successfully resync with the new trajectory slope.

Therefore, it can be seen from the figures that there is always a certain lag in the characterization and prediction of the actual degradation trajectory by the parameter adaptive model. For the more complex degradation laws contained in the data, better prediction methods are required for representation. The goodness-of-fit evaluation table after PF updating is shown in Table 3.

### 4.2. Experimental Results of Shielding Sleeve Life Prediction Based on the Hybrid Model

**Figure 12:**
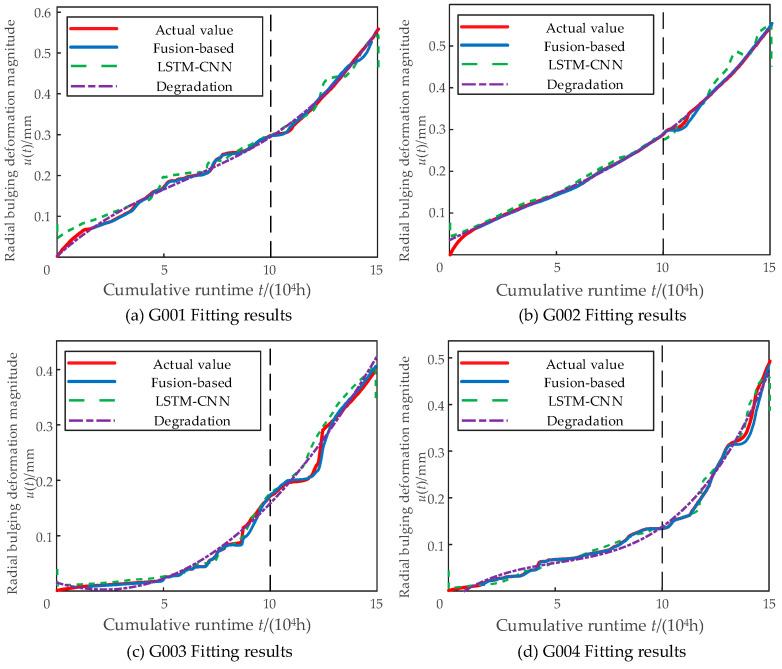
Failure prediction results of shield bulging based on hybrid model.

Life prediction of shielding sleeve bulging failure and wear failure is performed based on the hybrid model. Figure 12 presents the prediction results of shielding sleeve bulging failure, where the black dashed line denotes the dividing line between the training set and the test set.

The dashed line in the figure represents the boundary between the training set and the test set. It can be seen from the figure that the training set covers the first 10 × 10^4^ h of the shielding sleeve operation. During this period, the shielding sleeve is in the mid-early service stage, with a relatively smooth and slowly growing radial deformation curve. The deformation gradually accumulates from 0 to approximately 0.3 mm, showing typical stable early-stage degradation characteristics of the material. All methods achieve favorable prediction accuracy on the training set, among which the predicted curve of the proposed hybrid model is the closest to the actual degradation trajectory. It fully extracts the local features and long-term temporal dependencies in the time series, exhibits no underfitting, and captures the gentle nonlinear growth trend with high precision.

The test set corresponds to the late operation stage from 10 × 10^4^ h to 1.5 × 10^5^ h. It can be clearly observed that the slope of the curve increases during this stage, and the shielding sleeve gradually enters an accelerated degradation period. The radial deformation rises from 0.2 mm to about 0.5 mm, presenting a strong nonlinear acceleration characteristic. Compared with other methods, the hybrid model still maintains superior prediction accuracy by maximally utilizing the degradation trend information from the physical degradation model and the feature learning capability of the LSTM-CNN network. It can accurately characterize the nonlinear fluctuations in bulging deformation caused by possible operating condition variations or measurement errors during operation.

**Figure 13:**
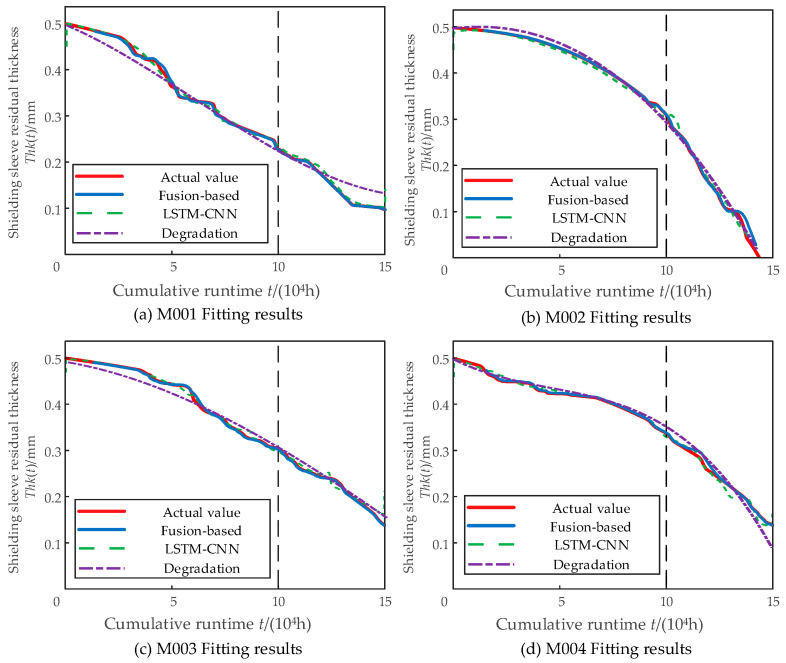
Prediction results of shielding sleeve wear failure based on hybrid model.

Figure 13 shows the prediction results of shielding sleeve wear failure. Similar to the prediction results of bulging failure, the proposed hybrid model yields extremely high prediction accuracy and excellent nonlinear mapping capability for both the training set and the test set, and achieves more stable and accurate RUL prediction for shielding sleeve failure data under different working conditions and degradation modes.

For model evaluation, the mean absolute error (MAE) is adopted, where the formula of MAE is given by:

(21) $$MAE=\frac{1}{n}\sum_{k=1}^{n}\left|\frac{y_{k}-{\hat{y}}_{k}}{y_{k}}\right|$$

The prediction performance of the proposed method is shown in Table 4.

Further analysis of Table 4 reveals the model’s performance differences across distinct prognostic stages. In the field of Prognostics and Health Management (PHM), evaluating near-failure prediction is critically more important than early-stage tracking. In our experimental design, the training set corresponds to the early and mid-term service stages (smooth degradation), while the testing set uniquely represents the near-failure accelerated degradation stage. As observed, the MAE and RMSE on the testing sets are generally slightly higher than those on the training sets. This accurately reflects the inherent difficulty of prognostics: as the shielding sleeve approaches failure, non-linear wear and complex internal perturbations dramatically increase, making the degradation trajectory inherently harder to predict. However, despite entering this highly non-linear near-failure stage, the hybrid model maintains a remarkably low MAE ranging from 0.0044 to 0.0201. This indicates that while early-stage prediction is nearly perfectly fitted, the model effectively arrests the typical exponential error growth in the near-failure stage, proving its robustness for critical pre-failure early warnings.

In summary, aiming at the limitations of single prediction models in the life prediction of shielding sleeves, the hybrid remaining useful life (RUL) prediction model based on Stacking ensemble learning proposed in this paper presents favorable performance in both prediction accuracy and reliability. For the physical model, the particle filter (PF) algorithm is introduced to realize the adaptive dynamic update of degradation model parameters, which effectively reduces the prediction deviation of fixed-parameter models. For the data-driven aspect, an LSTM-CNN deep learning network is constructed, integrating the capabilities of capturing long-term temporal dependencies and extracting local abrupt change features. The hybrid model combining data-driven methods and physical laws effectively overcomes the limitations of single models. It can achieve high-precision and low-uncertainty RUL prediction for shielding sleeves under various complex degradation modes, which fully verifies the effectiveness and strong generalization ability of the proposed method in the reliability assessment and health management applications of main pump motor shielding sleeves.

To fully quantify the accuracy of the remaining useful life prediction results, an uncertainty analysis is further conducted on the life prediction of the shielding sleeve. Considering the uncertainties in the degradation model, the initial assembly clearance and the creep coefficient of Hastelloy are replaced by deterministic weight values with probability distributions. In addition, the meta-learner in the hybrid life prediction model is replaced with a Bayesian Neural Network (BNN). Instead of learning fixed weight parameters, the network learns the distribution parameters of the weights. During each forward propagation of data, the network samples from these distributions, resulting in an output network that fluctuates due to parameter perturbations.

**Figure 14:**
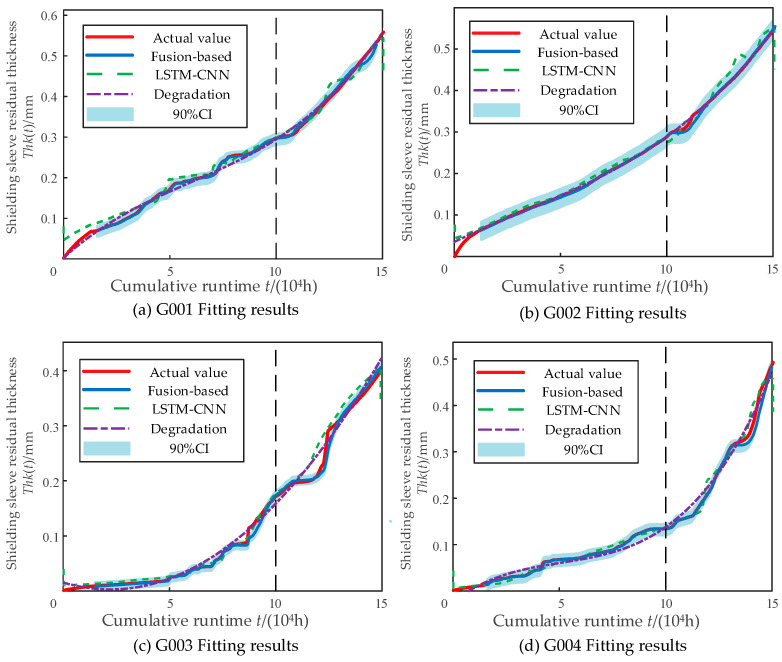
Prediction of Shield Bulge Failure Considering Uncertainty Quantification.

**Figure 15:**
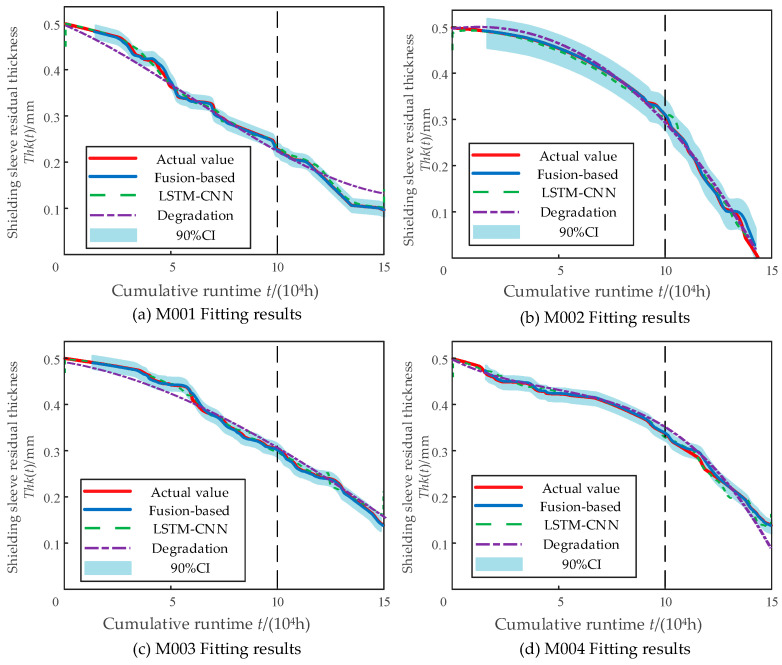
Prediction of Shield Wear Failure Considering Uncertainty Quantification.

Uncertainty analysis was performed on the bulging datasets G001–G004 and the wear datasets M001–M004. The ratio of the training set to the test set for each dataset was set at 2:1, with the prediction start time set at 10 × 10^4^ h and the initial learning rate set to 0.005. Figure 14 shows the prediction results of shielding sleeve bulging failure, and Figure 15 shows the prediction results of shielding sleeve wear failure. The black dashed line in the figures represents the boundary between the training set and the test set.

Based on the evaluation of MAE and RMSE, the Prediction Interval Coverage Probability (PICP) and Normalized Mean Prediction Interval Width (NMPIW) are used for evaluation. The expression of PICP is as follows:

(22) $$PICP=\frac{1}{n}\sum_{i=1}^{n}\mathrm{II}(L_{i}\leq y_{i}\leq U_{i})$$

In the formula, II denotes the indicator function, which equals 1 if the condition is satisfied and 0 otherwise. $L_{i}$ represents the lower bound of the prediction interval at time *i*, $U_{i}$ represents the upper bound, and $y_{i}$ is the *i*-th actual value. This indicator reflects the probability that the actual value falls between the upper and lower bounds of the prediction interval.

NMPIW is defined as:

(23) $$NMPIW=\frac{1}{n}\overset{\underset{n}{i=1}}{\sum }\frac{U_{i}-L_{i}}{R}$$

In the formula, *R* is the normalization factor, usually expressed as the variation range of $y_{i}$. A smaller NMPIW indicates a narrower prediction interval and less model uncertainty. Table 5 shows the evaluation metrics of the prediction results considering uncertainty.

It can be seen from the radial bulge deformation and residual thickness prediction diagrams of the shielding sleeve that the prediction results considering uncertainty quantification exhibit extremely high accuracy and stability under two different failure modes. The black dashed line in the diagram divides the data samples into the training set on the left and the test set on the right, and compares the prediction effects of the actual degradation trajectory, the hybrid model, the single deep learning model, and the traditional degradation model. From the bulge deformation from G001 to G004 and the decreasing trend of residual thickness from M001 to M004, it can be clearly observed that the prediction curve of the hybrid model constructed in this paper can fit the actual degradation trajectory more closely and smoothly compared with the single degradation model or the LSTM-CNN model. In addition, the 90% confidence interval output by the hybrid model maintains a narrow width throughout the prediction period. In G002 and M002, the confidence interval completely covers the true values, and there is no obvious error divergence or interval expansion in the later prediction stage. This method demonstrates favorable performance in terms of prediction accuracy and reliability.

### 4.3. Ablation Experiment

To verify that the performance improvement of the proposed method in this paper stems from the fusion of multiple methods rather than the contribution of a single method, ablation experiments and analyses of the proposed method are conducted on each dataset. Therefore, the following methods are compared:

(1) Hybrid model;
(2) LSTM-CNN;
(3) Degradation model.

By analyzing the data in Table 6, it can be seen that the proposed hybrid model method outperforms single models such as the degradation model and LSTM-CNN in both RMSE and MAE error metrics. By introducing uncertainty quantification, it can not only provide satisfactory prediction accuracy but also offer more interval information for condition-based maintenance compared with point prediction, thereby improving the engineering practicability of the prediction results.

## 5. Conclusions

This paper conducts a study on the life prediction of shielding sleeves under complex degradation modes. Aiming at the core problems of insufficient full-life-cycle degradation data and poor generalization ability of single prediction models, a remaining useful life (RUL) prediction method for shielding sleeves based on data augmentation and hybrid model is proposed. The specific contents are as follows:

1. A degradation data augmentation method based on Monte Carlo simulation is proposed, which generates virtual degradation trajectories with multi-source uncertainty via random sampling of working conditions and material parameters. This method effectively overcomes the shortage of training data for life prediction models and provides sufficient data support for the subsequent construction of prediction models.
2. The particle filter algorithm is introduced to realize the adaptive dynamic update of the parameters in the shielding sleeve degradation model. Compared with the traditional polynomial empirical degradation model, it effectively reduces the prediction deviation caused by fixed model parameters.
3. A hybrid model for remaining useful life (RUL) prediction based on Stacking ensemble learning is proposed. By combining the macro trend prediction capability of physical models and the micro feature capture capability of deep learning models within the ensemble learning framework, the accuracy and generalization ability of the remaining useful life prediction model for shielding sleeves are significantly improved.

## Figures and Tables

**Table 1:** Geometric Dimensions and Material Properties of the Shield Sleeve.

Parameter	Value	Parameter	Value
inner diameter/mm	409	heat capacity/(J/kg/K)	407
width/mm	0.5	density/(kg/m^3^)	8900
length/mm	1300	Young’s modulus/Pa	2.05 × 10^11^
air gap/mm	0.5	Poisson’s ratio	0.31

**Table 2:** Initial Value Table of Degradation Model Parameters.

Dataset Number	$\theta_{1}$	$\theta_{2}$	$\theta_{3}$	$\theta_{4}$
G001	0.0002	−0.0041	0.0475	0.0022
G002	0.0001	−0.0011	0.0248	0.0350
G003	1.5810	0.0025	−0.0115	0.0161
G004	0.0003	−0.0053	0.0347	−0.0227
M001	6.7556	−0.0015	−0.0211	0.5143
M002	−3.4470	−0.0022	0.0058	0.4897
M003	1.6518	−0.0012	−0.0090	0.5091
M004	−0.0002	0.0034	−0.0270	0.5048

**Table 3:** Table of Degradation Model Goodness of Fit after PF Update.

Dataset Number	Parameter Adaptive Update Model		Initial Degradation Model	
	RMSE	Adjust R^2^	RMSE	Adjust R^2^
G001	0.0351	0.9966	0.0413	0.9863
G002	0.0215	0.9988	0.0301	0.9878
G003	0.0482	0.9920	0.0597	0.9810
G004	0.0635	0.9902	0.0655	0.9847
M001	0.0521	0.9951	0.0607	0.9947
M002	0.0124	0.9986	0.0211	0.9786
M003	0.0326	0.9958	0.0342	0.9953
M004	0.0584	0.9908	0.0614	0.9875

**Table 4:** Prediction performance of hybrid models.

Dataset Number	Training Set		Testing Set	
	RMSE	MAE	RMSE	MAE
G001	0.0077	0.0067	0.0134	0.0101
G002	0.0023	0.0020	0.0075	0.0066
G003	0.0098	0.0088	0.0115	0.0099
G004	0.0134	0.0102	0.0247	0.0201
M001	0.0107	0.0084	0.0211	0.0154
M002	0.0077	0.0056	0.0058	0.0044
M003	0.0098	0.0077	0.0105	0.0078
M004	0.0072	0.0064	0.0170	0.0144

**Table 5:** Evaluation metrics of the prediction results considering uncertainty.

Dataset Number	Prediction Starting Point	PICP	NMPIW
G001	10 × 10^4^ h	0.9653	0.0455
G002	10 × 10^4^ h	1.000	0.0812
G003	10 × 10^4^ h	0.9327	0.0686
G004	10 × 10^4^ h	0.9155	0.0713
M001	10 × 10^4^ h	0.9584	0.0399
M002	10 × 10^4^ h	1.000	0.0681
M003	10 × 10^4^ h	0.9742	0.0520
M004	10 × 10^4^ h	0.9217	0.0614

**Table 6:** Ablation experiment results.

Dataset Number	RMSE			MAE		
	Degradation Model	LSTM-CNN	Hybrid Model	Degradation Model	LSTM-CNN	Hybrid Model
G001	0.0351	0.0215	0.0134	0.0294	0.0172	0.0101
G002	0.0215	0.0142	0.0075	0.0185	0.0121	0.0066
G003	0.0482	0.0256	0.0115	0.0415	0.0204	0.0099
G004	0.0635	0.0412	0.0247	0.0542	0.0345	0.0201
M001	0.0521	0.0384	0.0211	0.0436	0.0305	0.0154
M002	0.0124	0.0095	0.0058	0.0105	0.0081	0.0044
M003	0.0326	0.0198	0.0105	0.0275	0.0162	0.0078
M004	0.0584	0.0315	0.0170	0.0502	0.0264	0.0144

## Data Availability

The data presented in this study are available on request from the corresponding author.
